# Adaptation of Lipid Profiling in Depression Disease and Treatment: A Critical Review

**DOI:** 10.3390/ijms23042032

**Published:** 2022-02-12

**Authors:** Bruno Pinto, Tiago Conde, Inês Domingues, M. Rosário Domingues

**Affiliations:** 1 Centre for Environmental and Marine Studies, CESAM, Department of Chemistry, Santiago University Campus, University of Aveiro, 3810-193 Aveiro, Portugal; brunojpinto@ua.pt (B.P.); tiagoalexandreconde@ua.pt (T.C.); 2 Mass Spectrometry Centre, LAQV-REQUIMTE, Department of Chemistry, Santiago University Campus, University of Aveiro, 3810-193 Aveiro, Portugal; 3 Institute of Biomedicine—iBiMED, Department of Medical Sciences, University of Aveiro, 3810-193 Aveiro, Portugal; 4 Centre for Environmental and Marine Studies, CESAM, Department of Biology, Santiago University Campus, University of Aveiro, 3810-193 Aveiro, Portugal; inesd@ua.pt

**Keywords:** lipidomics, mass spectrometry, depression, major depressive disorder, oxidative stress, inflammation

## Abstract

Major depressive disorder (MDD), also called depression, is a serious disease that impairs the quality of life of patients and has a high incidence, affecting approximately 3.8% of the world population. Its diagnosis is very subjective and is not supported by measurable biomarkers mainly due to the lack of biochemical markers. Recently, disturbance of lipid profiling has been recognized in MDD, in animal models of MDD or in depressed patients, which may contribute to unravel the etiology of the disease and find putative new biomarkers, for a diagnosis or for monitoring the disease and therapeutics outcomes. In this review, we provide an overview of current knowledge of lipidomics analysis, both in animal models of MDD (at the brain and plasma level) and in humans (in plasma and serum). Furthermore, studies of lipidomics analyses after antidepressant treatment in rodents (in brain, plasma, and serum), in primates (in the brain) and in humans (in plasma) were reviewed and give evidence that antidepressants seem to counteract the modification seen in lipids in MDD, giving some evidence that certain altered lipid profiles could be useful MDD biomarkers for future precision medicine.

## 1. Introduction

Major depressive disorder (MDD) is a serious brain disorder characterized by periods of depressed mood, and sometimes combined with other symptoms, such as changes in appetite, sleep, fatigue, low self-worth and, in worst cases, suicidal tendencies [1]. This disease affects around 280 million people worldwide, which corresponds to 3.8% of the population, being one of the main causes of morbidity and disability in the world [2]. It is expected to become one of the main global burden of diseases by 2030 [3]. Due to the high prevalence of disability that translates in low productivity, and significant health and economic costs [4], MDD is considered one of the primary causes of the economic burden of disease worldwide [5]. Therefore, it is critical to tackle the incidence of this disease.

This disease is manifested in multiple forms and can be classified in different subgroups, depending on the presence of variable symptoms, their progression, or even the response to treatment [6]. There are 11 subtypes of MDD, that include melancholia, psychotic depression, anxious depression, or postpartum depression [7]. Diagnosis of depression is very subjective and often depends on symptoms and clinical observations [6,8,9,10], lacking specific biochemical parameters. Altogether this results in a high rate of misdiagnosis [11]. On the other hand, treatment of MDD includes a combination of pharmacological and non-pharmacological approaches and is highly dependent on the patient’s compliance. In addition, assessment of the therapeutic outcome is difficult due to lack of specific signs of prognosis and specific markers, and there is a high chance of reemission [12]. Moreover, the complex biochemical networks involved in the pathophysiology of this disease are poorly understood, which translates in a lack of biochemical markers that allow for a more accurate diagnosis and prognosis [13]. Recently, lipidomics has been assigned as an innovative tool to understand the pathophysiology and to discover new markers for non-communicable diseases [14]. This is being extended to MDD, as lipids have been highlighted as having a role in this disease and therefore have potential as biomarkers for diagnosis and prognosis [14,15].

Lipids are the main components of the brain, with roughly around 60% of dry weight [16]. They are involved in crucial brain functions, such as membrane composition, signal transduction, biological messenger functions, neuroendocrine function, and energy reserves [15], and also contribute substantially to biological processes associated with learning and memory [17,18]. Brain lipids are structurally diverse, and their composition is unique and quite different from other organs, comprising great diversity of classes belonging to phospholipids, sphingolipids, and triglycerides (TG). Moreover, phosphatidylethanolamines (PE) are the most abundant lipids in the brain, including both acyl and high content in plasmalogen PE species. They are followed by phosphatidylcholines (PC), phosphatidylserines (PS), phosphatidylinositols (PI), sphingomyelins (SM) and ceramides (Cer) [19]. The use of modern lipidomics approaches based on mass spectrometry (MS) allowed the understanding of brain lipidome, as well as to identify changes in brain and central nervous system lipidome related to several psychiatric diseases, namely schizophrenia and bipolar disorder [20,21], and some in MDD, as reviewed in this document.

The importance of lipids in the brain has led researchers to look at lipid dysregulation to understand MDD. Nowadays, it is recognized that lipid metabolism, in particular phospholipid and sphingolipid metabolism, is altered in psychiatric disorders, and may contribute to the etiology of these diseases. For instance, in MDD increased lipid peroxidation, derived from increased oxidative stress, as well as an inflammatory state, can induce changes in the lipid profile of the brain and in lipid metabolism, but also in lipids involved in signaling [22,23,24]. MDD treatment is also accompanied by changes in lipid metabolism [14,15].

As lipids are able to cross the blood-brain barrier, disturbance of lipid profiling associated with the oxidative stress and inflammation occurring in the brain could be mirrored in other matrices, e.g., blood plasma, which can be further evaluated by lipidomics [25]. Thus, lipidomics represents a promising approach to identify new biomarkers that could be used in the future for quantitative diagnosis or monitoring of response to treatment in the context of MDD [15], aiming for possible application in precision medicine, considering the individual diagnosis [26,27]. In this review, we will provide a state of the art on lipidomic analysis in MDD and in monitoring of antidepressant therapy. We will also present a perspective on the future potential of lipidomic as a tool in the context of MDD.

## 2. Causes and Effects of Major Depressive Disorder

MDD is a heterogeneous disorder resulting from multiple potential etiologies [9,11,28], but the pathophysiology of MDD remains unknown. The absence of explanation to describe depression’s origins and symptoms is a major barrier to fully understand its causes [11]. However, various hypotheses have been proposed to explain the pathophysiology of this disorder.

For instance, the biogenic amine theory of MDD suggests that norepinephrine (NE), serotonin (5-hydroxytryptamine, 5HT), and dopamine (DA), three important neurotransmitters, are decreased in this disease [29,30,31].The availability and actions of these neurotransmitters in the pre- and postsynaptic membranes of neurons are critical to most brain functions [29]. Their decrease may be due to impaired synthesis, release, or down-regulation of monoamine neurotransmitter receptor [32]. In fact, some studies have found associations between changes in 5HT levels, as well as NE and DA, and behavioral aspects including appetite, sleep, pain response, and circadian rhythm, all of which are linked to MDD [33,34].

Deficiency in monoamine transport proteins has also been associated with the development of MDD. They play important roles in neuron-neuron communication and monoaminergic transmission as facilitators of presynaptic reuptake, removing neurotransmitter molecules released into the synaptic cleft and returning them to presynaptic terminals, thus ensuring the continuity of neurotransmission and reducing degradation by the monoamine oxidase enzymes [35,36,37]. Moreover, depressed people have been reported to have problems with neurotransmitter-receptor coupling, which is usually caused by a decrease in the affinity of the receptor for neurotransmitters, a decrease in the number of receptors, and ineffective or abnormal transmission due to issues in the downstream signaling transduction [38,39].

Another hypothesis explains MDD through dysregulation of the hypothalamic-pituitary-adrenal (HPA) axis and suggests that hyperactivity of the HPA axis occurs, with elevated levels of cortisol. This may be due to reduced efficiency of central glucocorticoid receptor function, resulting in dysfunctional feedback from the HPA axis [35,37]. Expression of corticotropin-releasing factor (CRF), responsible for regulating the HPA axis, was found elevated in the hypothalamus of people with mood disorders. In addition, the loss of homeostatic control caused by chronically high CRF secretion may explain the reduced adrenocorticotropic hormone (ACTH) response to CRF, observed in people with depression [38].

The role of genetics in the development of depression is becoming more well recognized. Depression’s heritability does not appear to follow a traditional Mendelian pattern, according to genetic research [39]. Thus, it may explain why women’s heritability appears to be much larger than men’s, confirming higher incidence and prevalence of most types of depression in women [40]. Although several genes seem to be associated with development of depression, only a few genetic variants were described with significant association with MDD [41].

Mental health is also influenced by social and environmental variables. Smoking, lack of exercise, excessive alcohol use, and poor diets all lead to several comorbidities that can increase the risk of MDD [42]. Adverse childhood experiences, for example, have been linked to an increased risk of mental and physiological disorders later in life [43]. These negative events can have a direct impact on biological systems such as neuronal circuits, endocrine control, and immunological functions [44]. One study in monozygotic twins found that differences in the complexity of environmental experiences were related with the development of depression in the affected twin [40].

Recently, it has been recognized that oxidative stress plays an important role in the development of MDD [45]. When there is an imbalance between the oxidant and antioxidant processes, an excess production of reactive oxygen species (ROS) occurs. High accumulation of ROS can induce oxidative damage to biomolecules, as proteins and lipids, causing, for example, protein oxidation and cleavage and lipid peroxidation [25]. Furthermore, in studies linking chronic stress to altered immune function, several inflammatory cytokines have been implicated in the pathogenesis of depression [46]. Brain cells are highly susceptible to oxidative stress, as brain lipids are quite rich in polyunsaturated fatty acids (PUFA), which are key targets of modification by ROS [25,47]. This lipid oxidation can affect brain lipid profile and membrane lipid composition, and thus affect membrane structure, function, and properties [48]. It can also impact the assembly and function of membrane proteins and receptors and affect cell normal function and neurotransmission [49].

MDD is a complex disorder in which an interaction of genetic, environmental, immunological, and endocrine factors seems to contribute to its development. This results in an increase in oxidative stress [50] as well as in pro-inflammatory cytokines [51]. Together, these changes seem to affect the normal composition and function of the brain [52], affecting lipids due to peroxidation. These changes in lipids can be identified through lipidomic approaches, which could allow the identification of new biomarkers, needed for an effective screening and, consequently, to achieve an early treatment of MDD [42].

## 3. Lipidomics Approach

Lipids are an important class of biomolecules with different functions, such as membrane components, signaling molecules, and energy storage, and involved in vital cellular processes. They are a heterogenous group of biomolecules and can be divided in different classes according to their structure: (i) fatty acids, (ii) glycerolipids, (iii) glycerophospholipids, (iv) sphingolipids, (v) sterols, (vi) prenol lipids, (vii) saccharolipids, and (viii) polyketides [53,54]. These lipid categories can be divided into other classes and subclasses, increasing the diversity and complexity of the natural lipidome. Different cells and tissues have a specific lipidome composition, such as the brain or brain subregions [55]. Disturbances in the lipid profile can be associated with diseases and knowledge of the specific deviation of the lipidome in each disease is very promising for the identification of disease biomarkers, for diagnosis and prognosis, in addition to being important to unravel the pathophysiological processes associated with diseases and, thus, finding new and more efficient therapeutic approaches [56,57].

**Figure 1:**
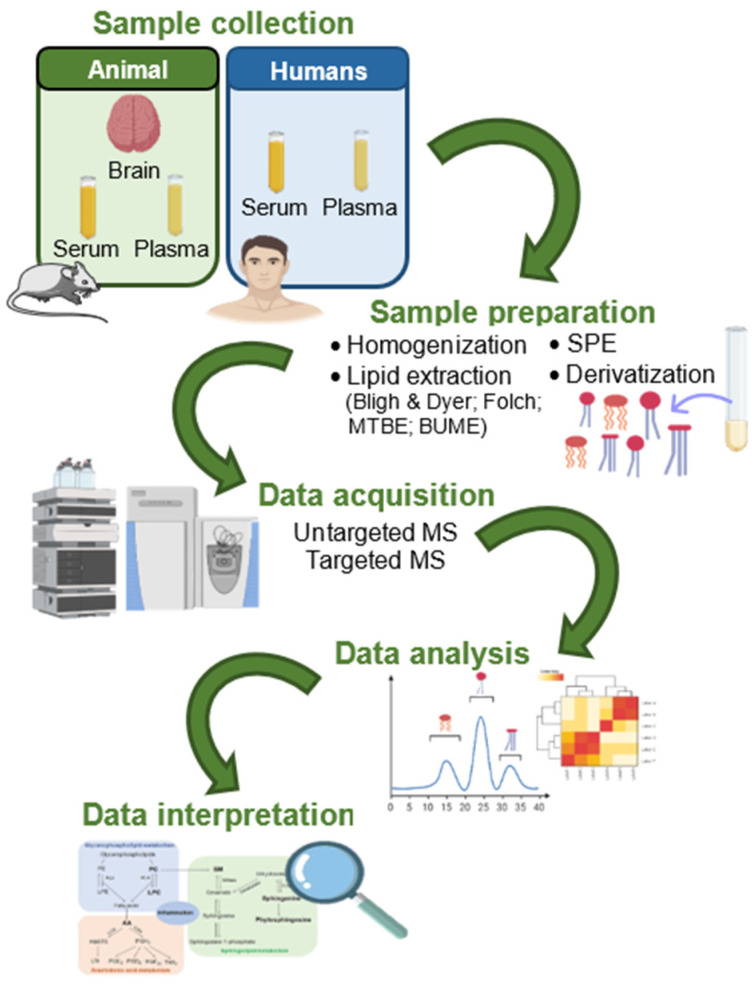
Workflow of lipidomics: from sampling, lipid extraction, LC-MS acquisition, data analysis and interpretation. Abbreviations: BUME, butanol/methanol; MS, mass spectrometry; MTBE, methyl *tert*-butyl ether; SPE, solid phase extraction.

The identification of the lipidome is currently performed by lipidomics approaches using methodologies based on MS. The lipidomics approach addresses the large-scale characterization, identification, and quantification of the molecular species of lipids in a specific sample. The general lipidomics workflow starts with sample collection, followed by lipid extraction and lipid extract analysis using MS-based methodologies. The obtained data are usually analyzed with the aid of statistical analysis bioinformatics tools to obtain the complete picture of the lipidome or its adaptation as a consequence of a disease (Figure 1), as recently reviewed [57,58].

Different types of samples can be used for lipidomics analysis, including cells, tissues or biofluids. After collecting the sample, the extraction of lipids is usually carried out with organic solvents. The most popular extraction methods use a combination of chlorinated solvents (chloroform or dichloromethane) with methanol and water, based on the Bligh and Dyer, and Folch methods, or a modified version of these. In these methods, a two-phase system is formed where lipids are extracted from the lower phase (organic), except for gangliosides which remain in the upper phase (aqueous) and need to be recovered using solid phase extraction (SPE). Other commonly used extraction methods are MTBE (methyl *tert*-butyl ether) and BUME (butanol/methanol). These latter methods have the advantage that the organic phase is in the upper phase and is therefore easier to recover [58].

Then, the lipid extracts can be analyzed directly by liquid chromatography coupled to MS (LC-MS) and tandem MS (LC-MS/MS) or analyzed by direct infusion-MS (DI-MS). LC-MS analysis can be performed using different types of columns, such as reverse phase (C18, C8 or C30 RP-LC-MS), normal phase (NP-LC-MS) or hydrophilic interaction (HILIC-LC-MS). RP-LC-MS allows separation of lipid molecules according to their fatty acyl chains, while separation in NP- or HILIC-LC-MS occurs according to their polar head groups, separating lipid class. Different mass spectrometers have been used in lipidomic approaches, but more recently high-resolution MS using Orbitrap, or quadrupole time-of-flight (Q-TOF) instruments are more frequently used because they allow accurate mass information to be obtained from molecular ions, as well as fragment ions, in addition to MS/MS interpretation [59]. DI-MS is usually used with target lipidomics based on shotgun approaches [60]. The identification of specific fragmentation for all identified ions is fundamental to pinpoint the structural characteristics of all molecular species of lipids and this is based on MS/MS analysis. The target fragmentation pathways allowed the obtainment of information on the polar head groups, as well as the composition and position of the fatty acyl chains. After unambiguous identification and annotation of the lipid molecules, quantification is achieved by peak integration. The huge amount of data generated in the LC-MS can be processed more efficiently using bioinformatics tools [57,61]. After processing the data, statistical analysis is performed and the integration of data will allow for the screening of patterns of variation in lipid profiles and abundances, comparing health and disease conditions [58].

Lipidomic approaches have been used to identify the lipidome from the brain and its subregions [55] in mice [62], as well as in different fluids (e.g., plasma, serum, cerebrospinal fluid) to identify characteristic markers for the diagnosis of brain conditions and disorders, such as MDD [14]. The following sections will describe and explain in detail the research studies found in the literature analyzing these changes.

## 4. Changes in Lipidome Associated with Major Depressive Disorder (MDD)

Published works have described changes in the lipidome of the brain or plasma of animal models of disease, as well as plasma and serum from humans with MDD. The results gathered in these works (Table 1) highlighted the changes in lipid classes and/or changes in lipid species/molecules. Moreover, these works did not identify variations in the lipidome according to the different subtypes of depression and most works excluded patients with other comorbidities from their studies. Only one work included patients with other diseases such as hypertension, diabetes, and high cholesterol [20], although no conclusions were drawn for each comorbidity together with MDD. The impact of these disorders in the lipidomic changes observed in MDD is of great interest and needs further research.

Plasma has been the most studied biofluid in this disease and is expected to reflect changes that occur in other tissues and organs, such as the brain. This is important in the case of MDD, where major modifications are expected to occur in the brain, that is inaccessible for molecular and biochemical analysis [63]. The accessibility of plasma makes it preferable to identify markers of lipids altered in MDD. The lipidomic characterization of depression was performed using common lipidomic approaches, based on MS technologies, although different extraction and acquisition methodologies were used in each publication, making an accurate comparison of results a challenge. In addition, different stimuli were used in the animal models’ studies to induce MDD, further hindering an accurate comparison.

**Table 1:** Lipid alterations reported in major depressive disorder.

Sample		↑	↓	Ref.
Rodents	Brain	PC; PE; OxCL	PI; CL	[64]
		Cer(16:0); Cer(16:1); Cer(18:1); Cer(22:1); Cer(26:1); LacCer(18:0); LacCer(24:0); LacCer(26:1)	PE(34:0); PE(34:1); PE(34:2); PE(36:0); PE(36:1); PE(36:2); PE(36:3); PE(36:4); PE(38:0); PE(38:1); PE(38:2); PE(38:3); PE(38:4); PE(38:5); PE(38:6); PE(40:4); PE(40:5); PE(40:6); PE(42:5); SM(16:0); SM(20:0); SM(22:0); SM(24:0); SM(26:0); dhSM(16:0); dhSM(16:1); dhSM(18:0); dhSM(18:1); dhSM(20:0); dhSM(22:0); dhSM(22:1); dhSM(24:0); dhSM(24:1); dhSM(26:0); dhSM(26:1); dhSM(26:2)	[65]
	Plasma	LPC(18:1); LPC(20:1); LPC-O(16:2); LPC-O(18:3)	PC(32:1); PC(36:4); PC(37:4); PC(38:4); PC(40:6); PC-O(36:4); PC-O(38:5); TG(58:12); TG(60:12); TG(62:13); TG(62:14)	[66]
Humans	Plasma	Cer(16:0); Cer(18:0); Cer(20:0); Cer(22:0); Cer(24:0); Cer(24:1); GluCer(24:1); LacCer(24:0)		[20]
		PC; PE; PI; LPC; LPE; LPI; PC-O; SM; TG; DG	PC-O; PE-O; SM	[67]
		PS(20:4); LPC(16:0); LPI(16:0); lysoPS(18:0); SM(24:0)	LPC(18:2); lysoPS(16:0); lysoPS(18:2); SM(18:2)	[68]
			PC-O(36:4); Cer(20:0); SM(16:0); SM(23:1)	[69]
	Serum	PS(34:2); LPI(16:0); LPI(18:2); TG(54:5); TG(54:6); TG(54:7); TG(54:8); TG(58:10); DG(32:0); DG(36:1); ChE(20:5)	PI(32:1); PI(32:2); PI(34:1); PI(34:2); PI(34:3); PI(36:2); PI(36:3); LPC(16:1); LPA(16:1); LPA(22:4); TG(48:2); TG(50:3); TG(50:4); TG(52:6); DG(36:8)	[70]

Cer, ceramide; ChE, cholesteryl ester; CL, cardiolipin; DG, diglyceride; dhSM, dihydrosphyngomielin; GluCer, glucosylceramide; LacCer, lactosylceramide; LPA, lysophosphatidic acid; LPC, lysophosphatidylcholine; LPE, lysophosphatidylethanolamine; LPE-O, alkyl-lysophosphatidylethanolamine; LPI, lysophosphatidylinositol; lysoPS, lysophosphatidylserine; PC-O, alkyl-phosphatidylcholine; OxCL, oxidized cardiolipin; PC, phosphatidylcholine; PE, phosphatidylethanolamine; PE-O, alkyl-phosphatidylethanolamine; PI, phosphatidylinositol; PS, phosphatidylserine; SM, sphingomyelin; TG, triglyceride.

The characterization of lipid changes in the brain tissue of animal models of MDD was only performed in two studies. Both works reported changes at different classes of lipids. For instance, Faria et al. (2014) analyzed changes of phospholipids at class-level, using thin layer chromatography (TLC) and LC-MS/MS, in the lipid extract obtained from the whole brain of mice with MDD after induction by an unpredictable chronic stress protocol, with a daily application of stressors. The obtained results revealed an increase in the relative abundance of the total content in PE and PC classes, and a decrease in PI and cardiolipin (CL) classes in TLC analysis. Analysis at molecular level through LC-MS/MS screening also revealed a significant decrease in CL species. Interestingly, this reduction was accompanied with an increase in oxidized CL (OxCL). The authors suggested this shift as a result from the unregulated oxidative stress that is observed in individuals with MDD [64].

On the other hand, Oliveira et al. (2015) studied the adaptation of the sphingolipid and phospholipid profile at molecular level of different brain regions, namely the prefrontal cortex (PFC), hippocampus, amygdala, and cerebellum, in a model of depression induced by chronic stress resulted from exposure to aversive stimuli. Significant changes were only observed in the PFC, namely increase in Cer and lactosylceramides (LacCer) species and decrease in SM, dihydrosphingomyelins (dhSM) and PE species (Table 1). Cer is generally increased in states of unregulated oxidative stress and in cell apoptosis, which can be indicators of cell death [71]. The authors suggested these changes to occur due to a dysregulation in the metabolism of sphingolipids through increased activity of acid sphingomyelinase (ASM), an enzyme that catalyzes the breakdown of sphingomyelin into ceramide and phosphorylcholine. Indeed, increased ASM activity is a hallmark of MDD metabolic alterations and is associated with the increase in ROS levels resulted from unregulated oxidative stress [72]. Moreover, increase in Cer can contribute to the progression of depression as they can alter the function of DA transporters, reducing transport of DA and increasing transport of 5HT [73]. Inhibition of ASM is already a promising therapeutic approach for MDD, and it targets the metabolism of sphingolipids, so several antidepressants seem to inhibit the upregulation of this enzyme in patients with MDD [74]. Overall, the observed changes in the brain lipids seem to be heavily marked by the oxidative status of MDD. The highlighted lipids, OxCL and Cer, were a result from the increase in ROS levels. They could comprise advantageous indicators for the diagnosis of depression. The differences observed between the lipid variations described in these studies could be due to the stressors used to induce chronic stress, and thus MDD, are different. In addition, these publications date of 2014 and 2015, suggesting that the data were not acquired with the most sensitive equipment, not allowing a coverage of lipids as efficiently as described in more recent studies.

Deviations in the plasma lipidome in MDD, in both the rat models with depression and in humans, were characterized. In both rodent and human plasma these studies reported an increase in lysophospholipids, such as lysophosphatidylcholine (LPC), lysophosphatidylethanolamine (LPE) and lysophosphatidylinositol (LPI), as well as alkyl-LPC (LPC-O) [66,67,68]. The observed increase in lysophospholipids may result from increased phospholipase A2 (PLA2) activity, responsible for the release of these lyso-lipids. Upregulation of PLA2 is often observed in inflammation, which is involved in the progression of MDD [75]. Furthermore, Chan et al. (2018) reported an increase in LPC(16:0), known to have pro-inflammatory activity, but also a decrease in LPC(18:2), which has been associated with anti-inflammatory activity, in human plasma [76]. This variation may indicate the prevalence of the pro-inflammatory state. Chronic inflammatory response is often associated with MDD patients [77]. Individuals suffering from depression have elevated levels of pro-inflammatory cytokines, such as tumor necrosis factor alpha (TNF-α) and interleukin-6 (IL-6), but also other mediators, such as chemokines, and this seems to contribute to the onset of this disease [46,77]. The use of antidepressants can reduce the levels of these pro-inflammatory mediators, which in turn may contribute to resolution of inflammation, and further attenuation of MDD [66,78,79].

However, other results were dissimilar between rodent and human plasma. In mice plasma it was reported a decrease in PC and TG [66], while in studies using human plasma authors reported an increase in Cer, PC, PE, diglyceride (DG) and TG, and decrease in PS and alkyl-PE (PE-O) and alkyl-PC (PC-O) [20,67,68,69]. Interestingly increase in Cer in human plasma was observed, probably occurring due to increase in ASM activity, as referred above [72]. Other highlighted changes were decrease in plasmalogens, such as PE-O and PC-O, which are well-known endogenous antioxidants. This can also be a result of increased oxidative stress observed in individuals with depression [67]. Moreover, plasmalogen species particularly from PE class, are quite abundant in the brain and are often described as having an antioxidant role, although this mechanism remains unclear [80,81]. Some authors suggest the alkenyl acyl lipids are more prone to oxidation, compared to other diacyl or alkenyl phospholipids [81]. Thus, decrease of plasmalogens may suggest a decrease of antioxidant defense in the brain.

A higher abundance of phospholipid species with high saturated fatty acids was verified in patients diagnosed with MDD, namely the lyso species LPI(16:0), lysophosphatidylserine (lysoPS) (18:0), and SM(24:0), which were proposed as a risk factor for depressive symptoms, through mechanisms of inflammation and increased oxidative stress. Furthermore, the lipid species containing linoleic acid (LA), lysoPS(18:2), LPC(18:2) and SM(18:2), were decreased. Although lower concentrations of LA are often associated with down-regulation of pro-inflammatory markers, this decrease can be explained by the increase of δ-6 desaturase activity in patients with depressive symptoms, which converts LA into arachidonic acid (AA), a precursor of pro-inflammatory products. This further supports the association between depression and chronic inflammation [68].

The lipid profiling of serum from patients with MDD revealed dissimilar results compared to the ones observed in the plasma. For instance, Kim et al. (2018) observed an increase in LPI species and decrease in PI species, while a relation of variations in DG and TG species could not be establish [70]. Although the authors did not elucidate on the increase in LPI and decrease in PI species, it is known that they may result from the increase in PLA2 activity. PI are membrane phospholipids which give rise to secondary messengers and are major contributors to transduction pathways [82]. They play an important role in secretory cascades and intracellular signaling pathways, and their decrease may cause a change in the signal transduction level of many hormones, neurotransmitters, and growth factors, which could contribute to the onset and progress of MDD [70].

Some TG species were increased, which although not discussed by the authors, may be a result of decrease in TG hydrolysis by lipoprotein lipase (LPL) enzymes, such as adipose triglyceride lipase (ATGL) and hormone-sensitive lipase (HSL) responsible for the breakdown of TG into DG and free fatty acids (FFA), to be further used as energy sources [83]. Dysfunction of these enzymes can lead to an increase in TG levels, suggesting low energy consumption, which may explain the increased fatigue observed in patients with MDD [84].

A decrease of lysophosphatidic acid (LPA) was also observed in the serum off patients with MDD. LPA is involved in several aspects of neurodevelopment, and reduction of LPA species affects neuronal plasticity. Decrease in LPA species may be related to the development and progression of neuropsychiatric diseases, such as MDD, and were already suggested as a fundamental pathological mechanism underlying depression [70]. On the other hand, a decrease in LPC with an unsaturated acid, namely LPC(16:1) was observed. Although no specific justification was proposed for this species, unsaturated LPC are often associated with anti-inflammatory activity, thus suggesting the predominant pro-inflammatory state [85].

In a similar trend to what was observed in the brain, the reported changes in the plasma and serum of rodents and humans seem to derive from chronic exposure to unregulated oxidative stress and pro-inflammatory response, as well as dysregulation of sphingolipid metabolism and PLA2 activity. Despite the similarities between the possible origin of these changes, no clear relationship between brain plasma from individuals with MDD was established. Nonetheless, the results gathered so far support the important role of lipids in MDD, although more work is needed to further this knowledge.

## 5. Modulation of the Lipidome after Antidepressant Therapy

Treatment of MDD is a challenge due to the complexity and the lack of biomarkers for its diagnosis. Moreover, the effectiveness of current drug therapy is highly variable, with high and significant rates of non-responders to antidepressant treatments [28,86]. The subjectivity of the symptoms and the absence of quantitative biochemical markers hinder an adequate assessment of therapeutic results. Therefore, great efforts are being made to develop new and valuable approaches to assess the effects of antidepressant strategies [87]. The use of lipidomics to monitor progression of therapeutics and for a better prognosis could translate into a more accurate strategy to treat patients with MDD and predict its outcome [86].

Some studies have assessed lipid alterations in animal models (rodents and primates) and in humans after administration of antidepressant treatment, and they were described in Table 2. Most work was performed using the brain and plasma of rodents treated with these drugs, while studies in humans were scarce. Interestingly, one study evaluated the impact of antidepressant drugs in the brain of primates, although these animals were not subjected to a stress protocol to induce MDD. Different classes of antidepressant drugs were also used in these studies, namely, tetracyclic, and tricyclic antidepressants, such as maprotiline and imipramine, respectively, and selective serotonin reuptake inhibitors (SSRIs), such as citalopram, escitalopram, paroxetine and fluoxetine, the latter one representing the most used drug in the overall studies.

The effect of maprotiline and paroxetine in the brain of rodents exposed to a stress protocol was performed by LC-MS and allowed identification of lipid variations at the molecular level. For instance, Lee et al. (2009) evaluated changes in the PFC, hippocampus, striatum and cerebellum regions of the brain, however significant changes were only observed in the PFC lipidome [89]. In their later work focused on the PFC lipidome, Lee et al. (2012) found a significant increase in LPC and LPE species after treatment, both saturated and unsaturated, and a significant decrease in PC, PE, plasmenyl-PC (PC-O/PC-P) and plasmenyl-PE (PE-O/PE-P) [88]. These alterations can be explained by the increased activity of calcium-independent phospholipase A2 (iPLA2), a class of PLA2 involved in the hydrolysis of phospholipids, with consequent release of fatty acids, such as docosahexaenoic acid (DHA) [95]. Phospholipid species in the brain are rich in DHA, which is crucial for the induction and maintenance of long-term potentiation and synaptic transmission [88,89]. Other changes included an increase in Cer and a decrease in SM species. The authors this variation to be a result of possibly increased ASM activity. However, increase in ASM activity is a hallmark of MDD and could suggested the inefficacy of maprotiline and paroxetine to revert this [73,96]. Further work is required to understand this variation in Cer and SM during treatment with these antidepressants.

Fluoxetine was used in two studies with rodents, in which they looked at the brain and plasma lipidome. For instance, Liu et al. (2021) analyzed the lipid changes, at molecular level, in plasma of rodents with MDD after treatment with fluoxetine. The authors reported an increase in PC, SM and TG species, and a decrease in LPC, AA and sphinganine (SA) species. The relation between the decreased LPC(16:0), and the increased PC(36:4), reflects the effect of fluoxetine as a reducer of PLA2 activity [79]. In addition, this species was previously reported in MDD (Table 1) and is known as having a pro-inflammatory action, thus its decrease could provide an anti-inflammatory effect towards pro-resolution of inflammation [79,95]. This is also corroborated by decrease in AA, an anti-inflammatory mediator [97]. In addition, increased levels of TG further demonstrated the capacity of fluoxetine to reverse the variations observed in MDD, and tackle common malnutrition observed during this disorder [79]. These results suggest that the treatment can modulate lipid metabolism in MDD and counteract the effect of this disease. On the other hand, Xue et al. (2020) evaluated the lipidome of the PFC and the hippocampus of rodents after the administration of fluoxetine combined with repetitive transcranial magnetic stimulation (rTMS) [90]. There were only significant alterations in the lipidome of the PFC with an increase in acylcarnitines (AcCa) and SM, and a decrease in PE, phosphatidic acid (PA), DG, TG and monoglycerides (MG) classes. The increase in SM could result from a decrease in ASM activity, suggesting a counteracting effect of fluoxetine. Moreover AcCa, responsible for stabilizing membranes, improving mitochondrial function, and increasing antioxidant activity, was also increased, thus suggesting an antioxidant effect of this treatment [98].

Changes in the serum lipidome of rodents with MDD after administration of imipramine revealed an increase of one species in each of the following classes: PA, SM, PE-O and DG; and a decrease in some species of SM, lysophosphatidylserines (lysoPS), PE, PE-O, alkyl-LPE (LPE-O) and TG. In response to imipramine treatment, long-chain PA was upregulated. These species are involved in phospholipid biosynthesis, membrane remodeling and secondary messenger signaling, in addition to regulating the immune response through macrophage recruitment [99]. On the other hand, lysoPS positively regulate inflammatory responses through activation of mast cells [100] and were decreased. The combined effects of PA and lysoPS suggest regulation of peripheral inflammation, revealing these species as potential markers of antidepressant treatment [101], as resolution of inflammation was reported to help boost behavioral responses to stress [102].

The analysis of plasma lipidome from humans after administration of citalopram and escitalopram revealed an increase in several PC and PC-O species, and to a lesser extent, LPC and SM species, and a decrease in PC(38:6), PC(36:4), PS and sphinganine-1-phosphate (SA1P) [92,93]. The authors suggested that the increase in PC species is responsible for increase in the activity of membrane proteins [92,93], e.g., carnitine palmitoyltransferase 1 (CPT1) which is important in the beta-oxidation of fatty acids [103]. Increase in PC-O, an important antioxidant phospholipid, could derive from a reduction in the unregulated oxidative stress and, consequently, in ROS levels. Both increase in PC and PC-O seem to counteract changes reported in MDD (Table 1), revealing a beneficial outcome of treatment with both antidepressants [92].

Finally, only one study reported lipid changes in the prelimbic cortex of the brain of healthy primates after treatment with fluoxetine. The effect of fluoxetine seems to affect mainly the fatty acyl residue composition, with changes being observed mostly in PUFAs [94]. More specifically, the authors reported that alkyl-hexosylceramides (HexCer-O) esterified in PUFA significantly increased after treatment. In contrast, a decrease in PE, LPE and FFA classes was observed and was suggested to derive from general changes in fatty acid metabolism as a potential adverse effects of fluoxetine treatment. In fact, the brain contains large amounts of long-chain PUFAs, which although not synthesized there, are transported across the blood-brain barrier [104]. Moreover, there is increasing recognition of the role played by PUFAs in the pathogenesis of psychiatric disorders, e.g., deficiency in long-chain omega-3 fatty acids that is a feature associated with MDD [105]. Although the authors have not explained the possible mechanism causing the treatment-induced decrease in PUFA abundance, they suggest that reduced liver biosynthesis may be one of the causes [94].

Interestingly, virtually no studies have compared changes in lipids in the brain and plasma/serum, and this should be investigated in the future. Furthermore, most of these studies did not compare MDD individuals after treatment with healthy control groups without depression, hindering the assessment of the drugs ability to neutralize changes associated with the pathophysiology of MDD, and how far the treatment with antidepressants can restore the healthy lipid profile. Moreover, only one study compared the results of lipid changes in the brain between two different antidepressants. On the other hand, there is a scarcity in the number of studies in humans, as well as a lack of assessment of the impact of these antidepressants in different subtypes of MDD. In addition, some studies have dissimilar and used different lipidomics platforms to achieve their results not allowing a true comparison between them. Despite the limitations of these studies, the findings clearly showed lipid membrane remodeling as an essential response to treatment of MDD, but also a key role in the modulation of signaling species, mostly responsible for enhancement or attenuation of inflammation, to drive the inflammatory response towards resolution.

## 6. Concluding Remarks and Future Perspectives

Among the various omics fields, lipidomics represents an innovating tool to characterize changes in the lipid profiles of biological samples and can be used in the search for biomarkers and molecular mechanisms underlying MDD, as well as the changes caused by pharmacological treatment with antidepressants, representing a promising approach to predict therapeutic outcomes.

Although few studies were performed in models of depression, lipids seem to play a vital role in the pathophysiology of this disease. In both rodents and humans, there was a clear increase in lysoglycerophospholipids (e.g., LPCs) and some sphingolipids, such as Cer, with pro-inflammatory activity, that seemed to derive from an increase in oxidative stress and cellular apoptosis, contributing to the chronic inflammatory state associated with MDD.

In the last two years, there was an increase in the number of studies on the modeling of the lipid profile after administration of antidepressant drugs, both in animal models (rodents and primates) and in humans. In general, there are differences between the lipidome of patients with MDD treated and untreated with these drugs. They seem to induce a counteracting action to tackle conditions associated with the pathophysiology of MDD, such as chronic inflammation, as these studies observed, among other lipid features, an increase in lipids with anti-inflammatory activity, possibly due to inhibition of the metabolism of sphingolipids and membrane phospholipids, and consequent reduction in inflammation.

However, among all the works, a reliability problem prevails mainly due to some flaws in the experimental design. In most studies there are no comparisons between healthy controls, MDD patients treated and untreated with antidepressant drugs, to assess the extent to which the treatment reverts to an overall healthy condition. Taking into account that most studies have used rodents, further work in other animal models is required to establish reliable lipid profiles for the diagnosis of MDD or monitoring of pharmacological therapy, e.g., in zebrafish. Furthermore, to date there is no research characterizing the profile of gangliosides in MDD, which are an important class of lipids highly abundant in the brain and essential for its development and plasticity. In addition, as depression is a multifactorial disease it is required to understand the difference between the lipid changes observed for each MDD subtype. Despite the contributions of existing studies, more research on this subject is required to better understand the changes in the lipid profile and metabolism that occur in this disease. This will be important to identify disease biomarkers to overcome the difficulty associated with the diagnosis of MDD and, consequently, to develop new monitoring and therapeutic strategies, particularly at the individual level, aiming at personalized medicine.

## Figures and Tables

**Table 2:** Modulations of lipidome after antidepressant therapy, comparing therapy with disease conditions. Lipid species in common between rodent brain and plasma are underlined. Lipid species in common between rodents and humans are in bold.

Sample		Antidepressant Treatment	↑	↓	Ref.
Rodents	Brain	Maprotiline	LPC(16:0); LPC(18:1); LPC(20:4); LPE(16:0); LPE(18:1)	**PC(38:6)**; PC(40:4); PC(40:5); PC(40:6); PC-O/PC-P(40:2); PE-O/PE-P(38:4); PE-O/PE-P(38:5); PE-O/PE-P(38:6); PE-O/PE-P(40:5); PE-O/PE-P(40:6); PE-O/PE-P(40:7)	[88]
		Maprotiline	**PC(30:0)**; PC(32:0); LPC(16:0); LPC(16:1); LPC(18:0); LPC(18:1); LPC(18:2); LPC(20:4); **PC-O(30:0)**; PC-O/PC-P(36:3); Cer(d34:1); Cer(d36:1); Cer(d38:1); Cer(d40:1); Cer(d42:1); SM(34:0)	PC(36:1); PC(38:3); PC(40:5); PC(40:6); PE(34:2); PE(36:5); PI(38:6); PI(40:4); PC-O/PC-P(40:2); PC-O/PC-P(42:5); PC-O/PC-P(42:6); PC-O/PC-P(42:7); PE-O/PE-P(36:4); PE-O/PE-P(40:4); SM(42:0); SM(42:1)	[89]
		Paroxetine	PC(32:0); PC(38:6); PI(38:6); PI(40:6); LPC(16:0); LPC(18:0); LPC(18:2); Cer(d36:1); Cer(d38:1); Cer(d40:1); Cer(d42:2)	PC(36:1); PC(36:3); PC(38:3); PC(38:4); PC(40:5); PC(40:6); PE(36:5); PE(38:5); PC-O/PC-P(40:1); PC-O/PC-P(40:2); PC-O/PC-P(42:5); PC-O/PC-P(42:6); PC-O/PC-P(42:7); PE-O/PE-P(36:4); SM(42:0); SM(42:1)	
		Fluoxetine + rTMS	SM; AcCa	PE; PA; TG; DG; MG	[90]
	Plasma	Fluoxetine	PC(34:0); PC(35:2); PC(36:4); PC(37:4); PC(38:2); PC(38:4); PC(38:5); PC(38:6); PC(40:6); PC(40:8); SM(d42:1); SM(d42:2); TG(50:2); TG(50:3); TG(52:4); TG(52:5); TG(54:4)	LPC(16:0); LPC(18:1); LPC(18:2); LPC(20:3); **LPC(20:4)**; SA; AA	[79]
	Serum	Imipramine	PA(44:4); PE-O(36:2); SM(d34:1); DG(38:2)	PE(44:12); PE-O(44:5); LPE-O(14:1); lysoPS(10:0); lysoPS(20:5); SM(d34:1); SM(d38:3); TG(42:2)	[91]
Humans	Plasma	Escitalopram	PC(36:2); PC(36:3)	PC(36:4); PS(34:2); PS(38:7); SA1P	[92]
		Citalopram/ escitalopram	PC(24:0); PC(28:1); **PC(30:0)**; PC(34:3); PC(34:4); PC(36:1); PC(36:6); PC(40:2); PC(42:2); PC(42:4); LPC(24:0); LPC(28:1); **PC-O(30:0)**; PC-O(34:2); PC-O(34:3); PC-O(36:3); PC-O(38:2); PC-O(42:2); SM(24:0)	**PC(38:6)**; **LPC(20:4)**; SM(18:1)	[93]
Primates *	Brain	Fluoxetine	HexCer-O **	PE **; LPE **; FFA **	[94]

AA, arachidonic acid; AcCa, acyl carnitine; Cer, ceramide; DG, diglyceride; FFA, free fatty acid; HexCer-O, alkyl-hexosylceramide; LPC, lysophosphatidylcholine; LPC-O, alkyl-lysophosphatidylcholine; LPE, lysophosphatidylethanolamine; lysoPS, lysophosphatidylserine; MG, monoglyceride; PA, phosphatidic acid; PC, phosphatidylcholine; PC-O, alkyl-phosphatidylcholine; PC-O/PC-P, plasmenyl-phosphatidylcholine; PE, phosphatidylethanolamine; PE-O, alkyl-phosphatidylethanolamine; PE-O/PE-P, plasmenyl-phosphatidylethanolamine; PI, phosphatidylinositol; PS, phosphatidylserine; rTMS, repetitive transcranial magnetic stimulation; SA, sphinganine; SA1P, sphinganine-1-phosphate; SM, sphingomyelin; TG, triglyceride. * The study was carried out in healthy animals, not subjected to a stress protocol. ** Composition-dependent variation in polyunsaturated fatty acids.

## Data Availability

Not applicable.
